# Single nucleotide polymorphisms in *ZNF208* are associated with increased risk for HBV in Chinese people

**DOI:** 10.18632/oncotarget.19669

**Published:** 2017-07-28

**Authors:** Hengxin Li, Jun Chen, RuiZhi Zhang, Ran Xu, Zhe Zhang, Le Ren, Qi Yang, Yumei Tian, Daxu Li

**Affiliations:** ^1^ Xi'an Center for Disease Control and Prevention, Xi'an, Shaanxi 710054, China; ^2^ Department of Pharmacy, The Ankang Central Hospital, Ankang, Shaanxi 725000, China; ^3^ Department of Stomatology, The Ankang Central Hospital, Ankang, Shaanxi 725000, China; ^4^ Department of Stomatology, The First Affiliated Hospital, Medical School of Xi'an Jiaotong University, Xi'an, Shaanxi 710061, China; ^5^ Department of Endocrinology, The First Affiliated Hospital of Xi'an Jiaotong University, Xi'an, Shaanxi 710061, China; ^6^ Key Laboratory for Tumor Precision Medicine of Shaanxi Province, The First Affiliated Hospital of Xi'an Jiaotong University, Xi'an, Shaanxi 710061, China; ^7^ Xi'an Mental Health Center, Xi'an, Shaanxi 710061, China

**Keywords:** ZNF208, SNPs, HBV, susceptibility, Chinese Han population

## Abstract

Single nucleotide polymorphisms (SNPs) in *ZNF208* may be associated with susceptibility to Hepatitis B virus (HBV). In the current study, we analyzed the association between *ZNF208* SNPs and risk of HBV in 242 HBV patients and 300 healthy subjects from the Xi'an area of Chinese Han Population. Of the five SNPs examined, rs2188971 (OR: 1.36, 95% CI: 1.04-1.76, *P* = 0.022), rs8103163 (OR: 1.40, 95% CI: 1.08-1.82, *P* = 0.010) and rs7248488 (OR: 1.38, 95% CI: 1.07-1.79, *P* = 0.014) were correlated with HBV susceptibility based on Chi-square tests. After the *P*-values were adjusted by Bonferroni correction, there only rs8103163 (*P* = 0.050) was slightly with increased HBV risk. Additionally, haplotype A_rs2188972_T_rs2188971_A_rs8103163_A_rs7248488_ (OR = 1.42; 95% C I, 1.10-1.85; *P* = 0.008) was found to increase susceptibility of suffering from HBV. These findings suggest that *ZNF208* polymorphisms may contribute to the development of HBV.

## INTRODUCTION

In the worldwide, there are approximately 240 million individuals who carried chronic hepatitis B virus (HBV), therefore, it remains a very serious health problem. [1, 2]. A Chinese survey of hepatitis B reported that Chinese people aged 1-59 years has a weighted prevalence of HBsAg with 7.2% [3], it showed that about 93 million people in China are infected with HBV. The risk factors of persistent viral infection include the following aspects: (1) host factors, contains sex, age, infection immunity and genetic variability; (2) virological factors, including viral gene mutation, gene type and viral load; (3) external factors, such as alcohol and chemotherapy [4]. Although the underlying molecular mechanisms have not yet been identified, we know that host genetic and viral factors are the crucial factors [5]. Some research studies have extensively investigated the relationship between candidate genes and the progression of HBV infection, including human leukocyte antigen system, cytokines, and killer cell immunoglobulin-like receptors [6–9]. But most of the candidate genes are still controversial in the immune response. The relationship between HBV infection and other host genetic diversities need further study. Persistent viral infection, HBV occurrence are influenced by immunological and host genetic factors [10–12], all of them are involved in host and virus interaction. Thus, in the investigation of disease pathology, researching the relationship between the host immune responses and gene polymorphisms may lead to new insights into HBV infection and the host responses. The future studies in different genes and their polymorphisms should gather useful information for better understanding and characterizing in HBV infection susceptibility.

Zinc finger (*ZNF*) genes belong to a large gene family, which has an estimated 500–600 members in the human genome [13]. The estimated 500 *ZNF* genes map to a variety of human chromosomes. Recently, one variant (rs8105767) within *ZNF208* gene was mentioned in a GWAS about the association with telomere length [14, 15]. The protein expressed belongs to Zinc Finger protein family [16, 17]. Through these structures, they can bind to DNA and further regulate gene transcription. The gene *ZNF208* may be one of the most crucial factors to suppress gastric cancer [18]. And another study showed *ZNF208* was involved in affecting the imatinib mesylate response to gastrointestinal stromal tumor [19]. Very large GWAS have identified some genes that are associated with interindividual variation in leukocyte telomere length, including SNPs in *CTC1, TERT, TERC, ACYP2, OBFC1, NAF1, ZNF208* and *RTEL1* [20, 21].

It is well known that single nucleotide polymorphisms (SNPs) can influence the disease progression following HBV infection. Therefore, detection of *ZNF208* polymorphisms could help us to predict HBV infection susceptibility and HBV-induced diseases outcome.

## RESULTS

A total of 242 patients with HBV and 300 healthy individuals were enrolled in the study. The participant characteristics are shown in Table 1. The mean age of the participants was 60.42 years in the control group and 50.04 years in the case group. There exist significant differences with *P* < 0.001 in gender, age, smoking, alcohol drinking between patients in the case and control groups. Therefore, any one of them may be regarded as confounding factors in the research between SNPs and HBV susceptibility in our case-control study.

**Table 1:** General characteristics among HBV cases and healthy controls

Variable	Case (242)	Control (300)	*P*
**Gender**			
Male	188	180	<0.001^*^
Female	54	120	
**Age, year (mean ±SD)**	50.04±12.048	60.42±5.143	<0.001^*^
**Smoking**			<0.001^*^
Ever	126	89	
Never	116	189	
**Alcohol drinking**			0.033^*^
Ever	90	79	
Never	152	199	

The PCR primers for the 5 selected SNPs, which were designed using the Sequenom MassARRAY Assay Design 3.0 Software, were listed in Table 2. Detailed SNP data and the associations between various SNPs and HBV risk are shown in Table 3. Our data indicated that all 5 SNPs investigated were in Hardy-Weinberg equilibrium in the control subjects (p > 0.05). We found there existed a correlation between three loci (rs2188971, OR: 1.36, 95% CI: 1.04-1.76, *P* = 0.022; rs8103163, OR: 1.40, 95% CI: 1.08-1.82, *P* = 0.010; rs7248488, OR: 1.38, 95% CI: 1.07-1.79; P = 0.014,) and HBV risk based on Chi-square tests. Rs2188972 and rs8105767 had no connection with HBV susceptibility. After the P-values were adjusted by Bonferroni correction, there only rs8103163 was slightly correlated with HBV risk (P = 0.050).

**Table 2:** Primers used in this study

SNP ID	1st-PCR primer sequence	2nd-PCR primer sequence	UEP sequence
rs2188972	ACGTTGGATGATTCAGAACCTGTGCAAAGC	ACGTTGGATGGGCTTGATTGGTCAAATGGC	GACTTCTCAAAGAACTAGAAA
rs2188971	ACGTTGGATGCACTAAATCAGACTGCTGAG	ACGTTGGATGCTCTTCAAAGATCTACTTC	TCCAAAACTAAAGTTGGCAAAA
rs8103163	ACGTTGGATGTTTTGGGCCAAAAACTTTG	ACGTTGGATGCCAGAAGATCTGAGATAAAG	cctGCCAAAAACTTTGGCATACT
rs7248488	ACGTTGGATGGTCACCAAAACACGTAATG	ACGTTGGATGACACACACAGACTCCTTCAC	gaggcCAGAATGGTCCACTAGAGA
rs8105767	ACGTTGGATGTAGTAGGCAGGGCCAGGCCA	ACGTTGGATGCTGCCCATATGGGCCATTTT	aAGTTACATCACCTGGGTATC

Note: SNP, single nucleotide polymorphism; PCR, polymerase chain reaction; UEP, unique base extension primer.

**Table 3:** Basic information of candidate SNPs in this study

SNP-ID	Alleles A/B	Gene	Band	Role	MAF-case	MAF-control	HWE-*P*	OR (95%CI)	*P*-value	*P*-adjusted
rs2188972	A/G	ZNF208	19p12	3'-UTR	0.516	0.468	0.817	1.21(0.95-1.54)	0.120	0.60
rs2188971	T/C	ZNF208	19p12	3'-UTR	0.343	0.277	0.774	1.36(1.04-1.76)	0.022^*^	0.11
rs8103163	A/C	ZNF208	19p12	Intron	0.351	0.278	0.886	1.40(1.08-1.82)	0.010^*^	**0.050**
rs7248488	A/C	ZNF208	19p12	Intron	0.350	0.280	0.669	1.38(1.07-1.79)	0.014^*^	0.060
rs8105767	G/A	ZNF208	19p12	-	0.268	0.271	0.187	0.99(0.75-1.29)	0.927	1

Note: SNPs, single nucleotide polymorphisms; MAF, minor allele frequency; HWE: Hardy-Weinberg equilibrium; OR, odds ratio; CI, confidence interval; A, minor alleles. B, major alleles. P-values were adjusted by the Bonferroni correction.

^*^: P-value <0.05 indicates statistical significance.

We further assessed the association between each SNP and HBV risk in an unconditional logistic regression analysis, which was performed using three models: dominant, recessive, additive and genotype model (Table 4).

**Table 4:** Associations between the SNP genotypes of ZNF208 and the risk of HBV

SNP-ID	Model	Genotype	Case	Control	OR (95%CI)	*P*	OR (95%CI)	*P*-adjust
rs2188972	Codominant	GG	57	86	1		1	
		GA	122	147	1.25(0.83-1.89)	0.285	0.97(0.58-1.61)	0.899
		AA	65	67	1.46(0.91-2.36)	0.118	1.18(0.65-2.15)	0.59
	Dominant	G/G	57	86	1		1	
		G/A-A/A	187	214	1.32(0.90-1.94)	0.163	1.03(0.64-1.67)	0.900
	Recessive	G/G-G/A	179	233	1		1	
		A/A	65	67	1.26(0.85-1.87)	0.244	1.21(0.73-1.98)	0.461
	Additive	-	-	-	1.21(0.95-1.54)	0.117	1.08(0.80-1.46)	0.601
rs2188971	Codominant	CC	100	157	1		1	
		CT	109	118	1.45(1.01-2.08)	0.044^*^	1.05(0.67-1.65)	0.837
		TT	26	24	1.70(0.93-3.13)	0.087	1.32(0.61-2.87)	0.482
	Dominant	C/C	100	157	1		1	
		C/T-T/T	135	142	1.49(1.06-2.11)	0.023^*^	1.09(0.71-1.68)	0.69
	Recessive	C/C-C/T	214	175	1		1	
		T/T	26	24	1.43(0.80-2.55)	0.234	1.29(0.61-2.72)	0.501
	Additive				1.36(1.04-1.77)	0.023^*^	1.11(0.79-1.55)	0.54
rs8103163	Codominant	CC	100	157	1		1	
		CA	114	119	1.50(1.05-2.15)	0.026^*^	1.10(0.70-1.71)	0.689
		AA	28	24	1.83(1.01-3.34)	0.048^*^	1.60(0.75-3.43)	0.22
	Dominant	C/C	100	157	1		1	
		C/A-A/A	142	143	1.56(1.11-2.19)	0.011^*^	1.17(0.77-1.79)	0.465
	Recessive	C/C-C/A	214	176	1		1	
		A/A	28	24	1.51(0.85-2.67)	0.163	1.54(0.74-3.18)	0.248
	Additive				1.41(1.09-1.83)	0.010^*^	1.20(0.80-1.54)	0.28
rs7248488	Codominant	CC	101	157	1		1	
		CA	114	118	1.50(1.05-2.15)	0.026^*^	1.09(0.70-1.70)	0.71
		AA	28	25	1.74(0.96-3.16)	0.068	1.51(0.71-3.19)	0.284
	Dominant	C/C	101	157	1		1	
		C/A-A/A	142	143	1.51(1.10-2.17)	0.013^*^	1.15(0.75-1.77)	0.507
	Recessive	C/C-C/A	215	175	1		1	
		A/A	28	25	1.43(0.81-2.53)	0.215	1.45(0.71-2.97)	0.313
	Additive				1.39(1.07-1.80)	0.014^*^	1.17(0.85-1.62)	0.336
rs8105767	Codominant	AA	132	154	1		1	
		AG	93	128	0.85(0.60-1.21)	0.36	0.77(0.50-1.21)	0.25
		GG	19	17	1.30(0.65-2.61)	0.454	2.10(0.85-5.20)	0.11
	Dominant	A/A	132	154	1		1	
		A/G-G/G	112	145	0.9090.64-1.27)	0.55	0.88(0.58-1.34)	0.555
	Recessive	A/A-A/G	225	182	1		1	
		G/G	19	17	1.40(0.71-2.76)	0.329	2.34(0.96-5.69)	0.061
	Additive				0.99(0.75-1.30)	0.926	1.04(0.74-1.48)	0.810

Note: OR, odds ratio; CI, confidence interval; SNP, single nucleotide polymorphism.

^*^: *P*-value <0.05 indicates statistical significance.

P-adjust were adjusted by the Bonferroni correction.

For rs2188971, in the codominant model, genotype “C/T” (95% CI, 1.01-2.08; *P* = 0.044) increased HBV risk by 1.45-fold; In dominant model, the genotype “C/T-T/T” (95% CI, 1.06-2.11; *P* = 0.023) increased HBV risk by 1.49-flod; In additive model, the allele “T” increased HBV risk by 1.36-fold (95% CI, 1.04-1.77; *P* = 0.023). We also observed another susceptibility SNP, rs8103163, in the codominant model, compared with genotype “C/C”, the genotype “C/A” (95% CI, 1.05-2.15; P = 0.026) and “A/A” (95% CI, 1.01-3.34; *P* = 0.048) increased HBV risk by1.50-fold and 1.83-fold, respectively; In dominant model, the genotype “C/A-A/A” increased 1.56-fold HBV risk (95% CI, 2.16 - 4.72); In additive model, the allele “A” increased HBV risk less than 1.5-fold (OR = 1.41; 95% CI, 1.09-1.83). The SNP rs7248488 also correlated an unfavorable effect with increased HBV risk in dominant model (OR = 1.51, 95% CI: 1.10-2.17, P = 0.013) and in the additive model (OR = 1.39, 95% CI: 1.07-1.80, P = 0.014). And in codominant model, the genotype “C/A” (95% CI, 1.05-2.15; P = 0.026) increased HBV risk by 1.50-fold.

**Figure 1:**
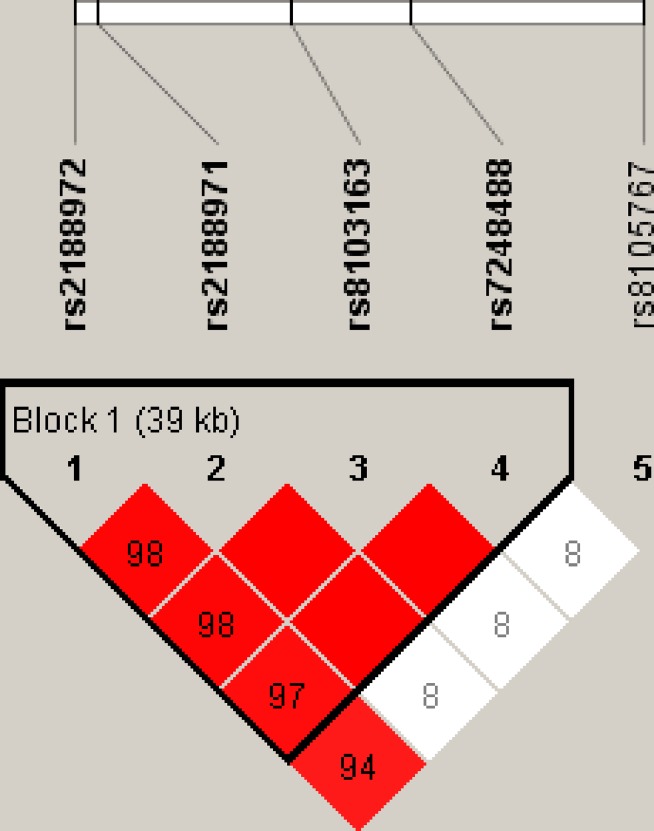
Linkage disequilibrium patterns of five SNPs in *ZNF208*

One block was researched in studied *ZNF208* SNPs by haplotype analyses (Figure 1). The results of the relationship between the *ZNF208* haplotype and the HBV susceptibility were shown in Table 5. Altogether there were three haplotypes and we only found haplotype A_rs2188972_T_rs2188971_A_rs8103163_A_rs7248488_ increase susceptibility of suffering from HBV by 1.42-fold (OR = 1.42; 95% CI, 1.10-1.85; *P* = 0.008).

**Table 5:** Haplotype analysis results in ZNF208

Haplotype	Frequency		OR (95% CI)	*P*	OR (95% CI)	*P*-adjust
	Case	Control				
rs2188972|rs2188971|rs8103163|rs7248488						
A TAA	0.3512	0.2767	1.42 (1.10-1.85)	0.008^*^	1.21 (0.87-1.68)	0.256
GCCC	0.4793	0.5283	0.82 (0.65-1.05)	0.112	0.92 (0.68-1.24)	0.565
ACCC	0.1694	0.1917	0.85 (0.62-1.76)	0.330	0.90 (0.60-1.36)	0.616

Note: SNP, single nucleotide polymorphism; OR, odds ratio; CI, confidence interval.

^*^p-value <0.05 indicates statistical significance.

P-adjust were adjusted by the Bonferroni correction.

## DISCUSSION

Our comprehensive analysis of *ZNF208* SNPs found that genotypes and haplotypes associate with increasing HBV risk. We detected three SNPs are related with HBV risk, rs2188971, rs8103163 and rs7248488. The other two *ZNF208* SNPs, rs2188972 and rs8105767, did not associate with HBV susceptibility. There is no studies reported that the association between *ZNF208* gene and HBV susceptibility in previously published studies. We studied the correlations between *ZNF208* SNPs and HBV susceptibility in Chinese Han population, and we got some significant results.

Telomere is the extreme end of chromosomal DNA, which is through the regulation of cell replication function to maintain genomic stability [22, 23]. Telomeres progressively shorten with the repeated cell division, because of the inability of DNA polymerase to complete the replication of the 3' end of linear DNA [24].

As we all know, the men are more easily to be infected with HBV than women [25]. In China, the HBsAg is significantly higher for males than females with a prevalence of 8.6% and 5.7% [26]. Reasons for sex discrepancy are complex, some research detected that sex hormones might interact with HBV infection process. Alcohol consumption, an environmental factor, has been implicated in HBV pathogenesis [27, 28]. However, the different outcomes of HBV infection cannot be fully explained the differences in environmental or viral factors [29]. To decrease bias of these confounding factors on effect estimates, we conducted the adjustment for sex, age, alcohol drinking and smoking. An association between age and acute HBV infection revealed that the risk of becoming a HBV carrier was correlated with age of the patient [29].

Zinc finger proteins (ZNFs), such as ZNF208, through this binding DNA to regulate gene transcription. A conserved protein motif, termed the Kruppel-associated box domain, mediates protein-protein interactions [30].

DNA binding of the encoded proteins is typically mediated by a ZNF motif that consists either of two histidines and two cysteines or four cysteines alone. The number of ZNF motifs is highly variable ranging from 2 to 40 copies in different members of the ZNF family. ZNF208 is a member of a large family of zinc finger proteins containing Kruppel-associated box domains, which serve as transcriptional regulators [31]. Unfortunately, there is a paucity of information about ZNF208 function in mammalian cells.

Recently, several case-control studies reported that *ZNF208*-associated SNPs were not associated with Chronic lymphocytic leukemia risk at P < 0.05 [32]. A meta-analysis found that seven loci involved in telomere biology, including rs755017 in *RTEL1*, rs9420907 in *OBFC1*, rs2736100 in *TERT*, rs10936599 in *TERC*, rs7675998 in NAF1, rs8105767 in ZNF208, and rs11125529 in ACYP2, and the subsequent large, case-control study identified the relationship between shorter LTL and increased risk of coronary artery disease in those of European population [21]. The genotype and allele frequency of rs8105767 was significantly different between the normal controls and the coronary heart disease [33]. In previously published studies, there had few research reported that the association between *ZNF208* gene and HBV susceptibility. In current study, we studied the correlations between five SNPs (rs2188971, rs8103163, rs7248488, rs2188972 and rs8105767) in *ZNF208* gene and HBV susceptibility in Chinese Han population.

Our study still had some limitations. Firstly, the statistical power of our study may be limited by the sample size and the future studies with a larger sample size should provide more valuable information. Secondly, some detailed clinical information of samples is incompleted and therefore further analysis to identify any associations between the SNPs and the clinical characteristics of these patients could not be done.

In conclusion, our findings revealed that *ZNF208* polymorphsims play complex roles in the development of HBV, and it could provide new evidences for the association between SNPs and haplotypes of *ZNF208* and the risk of HBV. This study offers new information on the relationship between *ZNF208* polymorphisms and HBV. Future functional studies are needed to confirm the correlation between the *ZNF208* gene and HBV pathogenesis, especially with respect to different ethnicities. Moreover, this study reveals the molecular markers associated with of HBV susceptibility and could therefore be used as diagnostic and prognostic markers for HBV patients in clinical study.

## MATERIALS AND METHODS

### Ethics statement

Our present study was approved by the Ethics Committee of the First Affiliated Hospital, Medical School of Xi'an Jiaotong University. Informed consent forms were signed by all participants. Consent was obtained from all the subjects participating in the present study.

### Study population

A total of 542 subjects (242 HBV patients and 300 healthy subjects) were recruited in our ongoing case-control study. We recruited the HBV patients (mean age ± SD = 50.04 ± 12.048) and 300 healthy subjects (mean age ± SD = 60.42 ± 5.143) from the Xi'an area of Chinese Han Population. All subjects were informed of the purpose of the study and the experimental procedures involved. We performed an uniform questionnaire in all of the subjects, the information include sex, age, alcohol consumption, family history of HBV infection, self-report of HBV transmission et al. All of the subjects were excluded from the study if they had a family history of HBV. The diagnostic criteria of HBV carriers were serum HBsAg positivity for over one year, normal liver function tests, and no clinical symptoms. The exclusion criteria were as follows: Non-HBV-related acute or chronic hepatitis; Liver cirrhosis or hepatocellular carcinoma; Mother to child transmission; Patients presenting HBsAg seroconversion post-CHB treatment.

### SNP selection and genotyping

We had selected five SNPs in *ZNF208* that had with minor allele frequencies (MAF) > 5% and were associated with HBV in the HapMap Asian population in current study. Venous blood samples (5 mL) were collected from each patient during a laboratory examination. We extracted genomic DNA from peripheral blood samples using the GoldMag-Mini Whole Blood Genomic DNA Purification Kit (GoldMag Ltd. Xi'an, China) according to the manufacturer's protocol. Sequenom MassARRAY Assay Design 3.0 Software was used to design primers for amplification and extension reactions [34]. SNP genotyping using the standard protocol recommended by the manufacturer was performed by Sequenom MassARRAY RS1000. Finally, Sequenom Typer 4.0 Software was used to perform the data management and analysis [34, 35].

### Statistical analysis

We used Microsoft Excel and the SPSS 17.0 statistical package (SPSS, Chicago, IL) to perform statistical analyses. All of the *p*-values presented in this study are two-sided, and p < 0.05 was used as the threshold of statistical significance. Chi-squared tests (categorical variables) and Student's t-tests (continuous variables) were used to evaluate the differences in the demographic characteristics between the cases and controls [36]. The Hardy-Weinberg equilibrium of each SNP was assessed in order to compare the expected frequencies of the genotypes in the control patients. All of the minor alleles were regarded as risk alleles for HBV susceptibility. Unconditional logistic regression models were used to estimate crude and adjusted odds ratios (OR) and 95% confidence interval (CI) for gender, age, alcohol drinking and smoking [37]. We used the web-based software SNP stats to test the associations between SNPs and the risk for PTB in four genetic models (genotype, dominant, recessive, and additive) [38].

The software platform (http://sampsize.sourceforge.net/iface/s3.html) was used for evaluating the statistical power of this case-control study. We used the Haploview software package (version 4.2) and SHEsis software platform (http://www.nhgg.org/analysis/) for analyses of linkage disequilibrium, haplotype construction, and genetic association at polymorphism loci [39, 40].
